# Supplementary material for: Modeling glioblastoma heterogeneity as a dynamic network of cell states
Source: Mol Syst Biol. 2021 Sep 16;17(9):e10105. doi: 10.15252/msb.202010105 (PMC8444284; doi:10.15252/msb.202010105)
Supplement: Supplementary file 6 — Source Data for Figure 5 [file MSB-17-e10105-s004.zip › Figure5A_sourcedata/GSEA_3017/hallmarks_stateE.GseaPreranked.1621934677158/HALLMARK_MYC_TARGETS_V1.html]

Details for gene set HALLMARK\_MYC\_TARGETS\_V1[GSEA]

|  || Dataset | state63017 |
| Phenotype | NoPhenotypeAvailable |
| Upregulated in class | na\_neg |
| GeneSet | HALLMARK\_MYC\_TARGETS\_V1 |
| Enrichment Score (ES) | -0.46381775 |
| Normalized Enrichment Score (NES) | -1.9709408 |
| Nominal p-value | 0.0012484394 |
| FDR q-value | 0.003607646 |
| FWER p-Value | 0.029 |
Table: GSEA Results Summary

  

Fig 1: Enrichment plot: HALLMARK\_MYC\_TARGETS\_V1      
 Profile of the Running ES Score & Positions of GeneSet Members on the Rank Ordered List

  

| PROBE | GENE SYMBOL | GENE\_TITLE | RANK IN GENE LIST | RANK METRIC SCORE | RUNNING ES | CORE ENRICHMENT || 1 | LSM2 |  |  | 289 | -0.256 | -0.2996 | No |
| 2 | RAN |  |  | 298 | -0.257 | -0.2917 | No |
| 3 | HSPD1 |  |  | 329 | -0.264 | -0.3074 | No |
| 4 | MCM5 |  |  | 337 | -0.265 | -0.2979 | No |
| 5 | HDAC2 |  |  | 348 | -0.268 | -0.2915 | No |
| 6 | IMPDH2 |  |  | 351 | -0.269 | -0.2763 | No |
| 7 | POLE3 |  |  | 367 | -0.273 | -0.2750 | No |
| 8 | HNRNPA2B1 |  |  | 391 | -0.279 | -0.2821 | No |
| 9 | SNRPG |  |  | 398 | -0.283 | -0.2704 | No |
| 10 | CDK2 |  |  | 422 | -0.290 | -0.2768 | No |
| 11 | KPNB1 |  |  | 445 | -0.298 | -0.2816 | No |
| 12 | HNRNPD |  |  | 456 | -0.301 | -0.2730 | No |
| 13 | MCM4 |  |  | 457 | -0.301 | -0.2535 | No |
| 14 | TRIM28 |  |  | 460 | -0.304 | -0.2361 | No |
| 15 | NOP56 |  |  | 483 | -0.309 | -0.2402 | No |
| 16 | PRDX3 |  |  | 489 | -0.310 | -0.2256 | No |
| 17 | ILF2 |  |  | 497 | -0.313 | -0.2130 | No |
| 18 | PA2G4 |  |  | 501 | -0.314 | -0.1960 | No |
| 19 | RANBP1 |  |  | 697 | -0.427 | -0.3818 | No |
| 20 | CDC45 |  |  | 773 | -0.521 | -0.4301 | Yes |
| 21 | RFC4 |  |  | 777 | -0.527 | -0.3993 | Yes |
| 22 | CDC20 |  |  | 793 | -0.562 | -0.3793 | Yes |
| 23 | USP1 |  |  | 806 | -0.575 | -0.3553 | Yes |
| 24 | DEK |  |  | 812 | -0.591 | -0.3225 | Yes |
| 25 | MCM7 |  |  | 817 | -0.599 | -0.2881 | Yes |
| 26 | PCNA |  |  | 832 | -0.640 | -0.2621 | Yes |
| 27 | BUB3 |  |  | 834 | -0.641 | -0.2217 | Yes |
| 28 | RRM1 |  |  | 866 | -0.749 | -0.2072 | Yes |
| 29 | CCNA2 |  |  | 872 | -0.770 | -0.1628 | Yes |
| 30 | KPNA2 |  |  | 895 | -0.858 | -0.1314 | Yes |
| 31 | DUT |  |  | 903 | -0.887 | -0.0817 | Yes |
| 32 | TYMS |  |  | 917 | -0.965 | -0.0334 | Yes |
| 33 | MAD2L1 |  |  | 921 | -0.990 | 0.0274 | Yes |
Table: GSEA details [plain text format]

  

Fig 2: HALLMARK\_MYC\_TARGETS\_V1: Random ES distribution      
 Gene set null distribution of ES for **HALLMARK\_MYC\_TARGETS\_V1**
